# MDGs and NTDs: Reshaping the Global Health Agenda

**DOI:** 10.1371/journal.pntd.0002529

**Published:** 2013-12-12

**Authors:** James Smith, Emma Michelle Taylor

**Affiliations:** 1 Centre of African Studies, University of Edinburgh, Edinburgh, United Kingdom; 2 Department of Geography, Environmental Management and Energy Studies, University of Johannesburg, Johannesburg, South Africa; 3 Centre of African Studies, University of Edinburgh, Edinburgh, United Kingdom; Centers for Disease Control and Prevention, United States of America

The United Nations (UN) Millennium Development Goals (MDGs) expire in 2015. A high-level panel, appointed to discuss the global development agenda post-2015, reported back in May 2013 with its recommendations. These are likely to prove extremely important for determining the global health budget over the coming decade. Who the “winners”—those who will benefit from UN endorsement and enhanced funding—and the “losers”—those not receiving such recognition or resources—will be in the new agenda is not yet decided, but certain parties hope that this time around NTDs will gain a special mention.

The MDGs, established in 2000, gave a new prominence to the health issues affecting the poor. However, the spotlight they provided was restricted and derived from a top-down process of deliberation, rather than informed by inclusive analysis and/or a thorough prioritisation of development needs. Subsequently, the narrowly focused and largely sector-specific MDGs left gaps in coverage and failed to realise synergies between the foci covered by the goals (education, health, poverty, and gender) [1]. MDG 6 in particular—“combat HIV/AIDS, malaria and other diseases”—sidelined many of the communicable and non-communicable diseases that perpetuate the cycle of poverty in developing countries. And yet, the very act of naming HIV/AIDS and malaria raised the profile of these diseases immeasurably. It stimulated a reconfiguration of official development assistance for health. Global health initiatives such as the Global Fund to Fight AIDS, Tuberculosis and Malaria (GFATM) and the President's Emergency Plan for AIDS Relief (PEPFAR) ushered in an era of vertical aid on an unprecedented scale and diverted resources away from existing health programmes [2]. In this funding climate, diseases were pitted against one another and advocacy groups were left to argue that it was their disease being referred to in the ambiguous wording “other diseases.” In this respect, the case of tuberculosis is instructive; the success of the tuberculosis campaigning was such that it is now widely assumed that it too received a special mention in the MDG 6. Of course, parallel to this misapprehension, tuberculosis was considered so central to the GFATM that it was even incorporated into the name; the extent to which this is due to lobbying or to the specific interactions between HIV/AIDS and tuberculosis has not been established. Clearly, however, effective networking and alliance building can blur the boundaries of the MDGs and raise the profiles of diseases.

## Establishing the NTDs

The 17 NTDs identified by the World Health Organization (WHO) represent some of MDG6's “other diseases.” This neglected tag stems from the disparity between the attention and funding these diseases receive (0.6% of official development assistance for health) and their catastrophic impact in terms of disability-adjusted life years (DALYs) [3], [4]. The perception of the neglect of these diseases is exacerbated when one considers the importance of the role the NTDs play as drivers and indicators of poverty [5], undermining efforts to meet the targets of virtually all the other goals [6], [7]. One could even argue that their ubiquity as relatively invisible cross-cutting drivers of poverty has paradoxically limited concerted efforts to focus on them.

The case for including NTDs in the post-2015 agenda has been building since their ostensible omission from MDG 6, which served as a call to arms for a group of concerned stakeholders, who have since contributed to a series of landmark initiatives that have placed NTDs firmly on the international agenda.

The term “neglected diseases” was coined by Kenneth Warren of the Rockefeller Foundation in the early 1980s through his Great Neglected Disease Initiative. The concept was revived in 2003, when the first of two WHO/Deutsche Gesellschaft für Technische Zusammenarbeit (GTZ) meetings was convened to float the idea that these diseases should be taken forward as a group, because they shared considerable geographical overlap, were in many cases syndemic, and could better be addressed by creating synergies between existing vertical programmes [8], [9]. Also in 2003, the Drugs for Neglected Diseases *initiative* (DND*i*) and the Foundation for Innovative Diagnostics (FIND) were established. In 2005, a second WHO/GTZ meeting was held, WHO set up a department for Neglected Tropical Diseases, and a group of previously obscure “parasitic diseases” secured a mention in the Commission for Africa Report [10]. In 2006, the Global Network for Neglected Tropical Diseases (focusing on the seven most prevalent NTDs) was formed, and integrated NTD control was awarded a Congressional earmark in the United States. In 2007, the specialist journal *PLOS Neglected Tropical Diseases*—the result of a collaboration between the Bill & Melinda Gates Foundation and the Public Library of Science—published its first article online. In 2010, WHO released its *First Report on the NTDs*, pinning down the 17 focal diseases we now know by the shorthand “NTDs” [11]. In 2012, WHO followed up that landmark document with a roadmap for action [12], and the *London Declaration on Neglected Tropical Diseases* was endorsed by a wide range of stakeholders [13]. In May 2013, a milestone WHO resolution on the NTDs was adopted [14].

Alongside these events, the NTDs secured unprecedented funding from traditional and non-traditional sources. Bilateral donors, the UK's Department for International Development (DFID), and the US Agency for International Development (USAID) remain important. However, in terms of volume, it has been the philanthropic contributions of the Bill & Melinda Gates Foundation (pledging US $363 million over the next five years) driving research agendas and the in-kind drug donations provided by the pharmaceutical industry (worth between US $2–3 billion annually [15]) that have shaped mass drug administration programmes that have proved decisive in changing the fortunes of several of the NTDs. The galvanising force behind this change in fortune for the NTDs has been new forms of partnership between the public and private sectors.

Already the fruits of the public-private partnership approach are being felt, with gains in NTD control providing hope that elimination may be a possibility for many of the diseases [16], [17]. The success has been such that the WHO Secretary General, Dr. Margaret Chan, recently referred to the story of the NTDs in the 21^st^ century as one of “rags to riches” [18]. In this extraordinary reversal of fortunes, the centrality of branding cannot be downplayed [15], [19]; where once 17 disparate diseases (caused by different pathogens and with varying susceptibility to control or elimination) were easily ignored, under the rubric of NTDs, they have become a clarion call for pulling the world's “bottom billion” out of poverty [20].

## Do the MDGs Really Matter?

Given the recent meetings of the high-level forum to discuss the post-2015 agenda and the high-profile debate around both the success or otherwise of the MDGs, and what might supersede them, it is significant to reflect that the strides made in NTD control in the first decade of the 21^st^ century were made despite the diseases' effective omission from the MDGs. Does this mean that getting onto the post-2015 agenda is immaterial to the NTDs? In this light, it is instructive to look at exactly who has endorsed the London Declaration on NTDs. The bulk of signatories are pharmaceutical companies, DND*i*, and the Bill & Melinda Gates Foundation; the only traditional donors to sign are USAID, DFID, and the World Bank. It is possible then that global health post-2015 might be driven by new sets of partnerships and actors. That said, the emulation of MDG-style time-bound targets in both the WHO Roadmap and the London Declaration's “scorecard” format suggests the NTD community has been deeply influenced by the UN's original goals. In addition, it is no coincidence that the notion of the NTDs included as part of a “gang of four” expanding the current “big three”, based on their comparable burden of DALYs, was floated in a series of policy papers co-authored by three scientists intent on influencing the debate around priorities in the wake of the original MDG decision [6], [21].

These scientist-influencers—Peter J. Hotez, David Molyneux, and Alan Fenwick (with Lorenzo Savioli and others)—helped to develop the rationale for the NTDs to be viewed as an aggregate group. Moreover, they persuasively argued that mass drug administration for the seven most prevalent NTDs represented one of the “best buys” in global public health, presenting the evidence to accompany their argument in the form of statistics, case studies, and pricing scenarios (US $0.50–0.79 per person, per year) [8], [21]. And yet, despite developing a compelling business case for the NTDs to go it alone in the wake of their effective omission from the MDGs, the policy papers released post-2000 were explicit that the ideal scenario was for NTD control to be integrated into broader health programmes, specifically those for the “big three” [22], [23]. Aware of the financial benefits that flow through the MDG name check, the authors were open in their desire to see an NTD focus incorporated into global health initiatives, the GFATM and PEPFAR, or better yet, an initiative solely focused on the NTDs [24].

Finally, it is pertinent that the NTD policy papers repeatedly depicted the NTDs as direct hindrances to the attainment of the MDGs; conversely, tackling them head-on was portrayed as directly beneficial to seven out of the eight goals [6]. In short, the NTD lobby has never been disinterested in the MDGs. They are fully cognisant of the ramifications of the NTDs' effective omission from MDG 6. They have spent the last decade trying to counter the ill effects, drawing on new partners and new models to ensure a stake in the post-MDG policy process, regardless of the direction that might take.

## Post-2015

The MDGs served to entrench an established tendency for donors to work in disease silos and have been critiqued accordingly. However, whatever hope there is for drastic change in the post-2015 development agenda, the MDG legacy will not be easily overturned (GFATM, vertical programmes, institutions, and long-term commitments will not be willingly dismantled). In the revised post-2015 agenda, there is at least the sense that we must move away from mortality-based ways of prioritising global health needs. In this respect, the NTD lobbyists such as the WHO, Global Network, and indeed *PLOS Neglected Tropical Diseases* itself, have been massively influential, breathing new life into DALYs, and, by turn, opening the door for more nuanced indicators of good health to be accepted—quality-adjusted life years or even average life expectancies [4]. Indeed, the recently released a report of the high-level panel on the post-2015 development agenda (mentioned in the introduction) includes an “illustrative goal” for health that will “ensure healthy lives” and explicitly names the NTDs alongside HIV/AIDS, tuberculosis, malaria, and non-communicable diseases [25]. Hopefully this constellation of actors, the emergence of new perspectives on health, and the publication of the New Global Partnership Report will convince member states to transform into stakeholders.

One “advantage” that the NTDs may have in a more enlightened and nuanced post-MDG era (with regards to development as well as to health), is that, by their very nature, they “undermine healthy lives” and cut across and threaten to undermine multiple silos of MDGs. This suggests a potentially fruitful bifurcated approach where focusing on NTDs can help make concrete inroads into reaffirmed or tweaked post-2015 MDGs, or NTDs can be used to articulate a set of goals that do not represent silos as targets to be met, but rather represent the strengthening of the institutions we need to manage the complex social, economic, environmental, and health systems that interact to shape future development. The former approach serves to underline the imperative to deal with NTDs if we are to make further progress. The latter, and preferable, approach can use the NTDs as a prompt to think of the goals not only as a clarion call, but also as an approach to the future of international development.
